# Biomass pellets for power generation in India: a techno-economic evaluation

**DOI:** 10.1007/s11356-018-2960-8

**Published:** 2018-08-24

**Authors:** Pallav Purohit, Vaibhav Chaturvedi

**Affiliations:** 1 International Institute for Applied Systems Analysis (IIASA), Laxenburg, Austria; 2 Council on Energy, Environment and Water (CEEW), New Delhi, India

**Keywords:** Biomass pellet, Agricultural and forestry residues, Biomass co-firing, Levelized cost of electricity, Employment generation

## Abstract

Modern bioenergy is being recognized as an increasingly important low-carbon resource by policy-makers around the world to meet climate policy targets. In India also, there is a clear recognition of the significant role of bioenergy in electricity generation as well as in other applications. In this study, a preliminary attempt has been made to assess the techno-economic feasibility of biomass pellets-based power (BPBP) generation in India. Surplus availability of biomass feedstock from agriculture and forestry/wasteland sector is estimated at 242 million tonnes (Mt) for 2010–11 and is expected to rise to 281 Mt in 2030–31 due to increased crop production and associated waste/residue availability. In terms of related capacity, the potential of BPBP projects is estimated at 35 GW for 2030–31. The associated carbon dioxide mitigation potential resulting from the substitution of coal is estimated at 205 Mt in 2030–31 if the entire biomass surplus is to be diverted for power generation. The levelized cost of electricity is estimated at €0.12 per kWh for BPBP projects as compared to €0.10 per kWh for imported coal based power plants. For states with the lower tariff for biomass power, the break-even price of carbon for BPBP projects is estimated at €18 per tonne. Additionally, BPBP projects will generate employment of more than 5 million person-months in the construction of biomass power plants and over 200,000 full-time employments in the operation of BPBP plants and in the production of biomass pellets.

**Electronic supplementary material:**

The online version of this article (10.1007/s11356-018-2960-8) contains supplementary material, which is available to authorized users.

## Introduction

Bioenergy (including traditional biomass) is the largest renewable energy source with 14% out of 18% renewables in the energy mix (WEC 2016) and supplies 10% of global energy supply (IEA 2016). Most of this is consumed in developing countries for cooking and heating, using traditional cook stoves, with considerable impact on human health (indoor air pollution) and on the environment (Rao et al. 2012; Yamamoto et al. 2014). Modern biomass is produced in a sustainable manner for electricity/heat production and biofuel for transport sector whereas traditional biomass is produced in an unsustainable way and it is used as a non-commercial source in inefficient stoves (Goldemberg and Coelho 2004). In 2010, the share of bioenergy as a whole accounted for 12% of the world’s total final energy consumption in which 9% came from traditional sources and 3% from modern bioenergy (IRENA 2016). Therefore, a rapid increase in modern biomass use is essential in order to achieve the international targets to double the global share of renewables by 2030. Global bioenergy production could increase to 180 EJ in 2050 (compared to 50 EJ in 2010 under business-as-usual scenario) under a 2.6 W/m^2^ climate policy scenario with the imposition of a carbon tax on both the fossil fuel and land-use sectors (Chaturvedi et al. 2015).

**Fig. 1:**
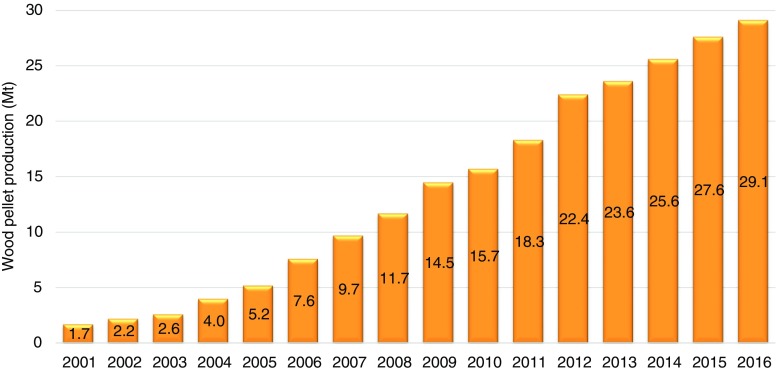
Global production of biomass pellets (source: Dwivedi et al. 2014; WBA 2014; FAO 2017; REN21 2017)

Efforts to reduce the poor handling properties of biomass feedstock (i.e., its low bulk density and the resulting low volumetric energy density and inhomogeneous structure) have led to increasing interest in the development of biomass briquetting and pelletization (Tripathi et al. 1998a; Holm et al. 2011; Stelte et al. 2012; Toscano et al. 2014; Hansson et al. 2015; Liu et al. 2016; Shone and Jothi 2016). When compared to other types of modern bioenergy, the pellet sector is one of the fastest growing. In 2016, 29.1 million tonnes (Mt) of pellets were produced worldwide in more than 800 plants with an individual capacity of over 10,000 tonnes (FAO 2017). The annual growth of biomass pellet production has been close to 20% over the last decade (WBA 2014) and has increased considerably in recent years, mainly due to the demand created by policies and EU’s bioenergy use targets (Dwivedi et al. 2014). Figure 1 presents the global production of biomass pellets (Dwivedi et al. 2014; WBA 2014; FAO 2017; REN21 2017). The top producers were the EU (49%), the USA (22%), Canada (10%), Vietnam (5%), and Russia (3%). In Europe, the EU 2020 policy targets for renewable energy sources and greenhouse gas (GHG) emissions reduction are among the main drivers for the large-scale utilization of wood pellets (Sikkema et al. 2011). Wood pellet production and export from southeast of the USA have doubled since 2011 (Prestemon et al. 2015; Hanssen et al. 2017) primarily due to EU demand (Abt et al. 2014) making the region one of the largest global wood pellet suppliers to the EU (Hoefnagels et al. 2014).

In developing countries, decreasing availability of fuelwood has necessitated efforts for more efficient utilization of traditional biomass feedstocks. For example, about 25% of total energy consumption is estimated to be met from various biomass resources (i.e., agri-residues, animal dung, forest waste, fuelwood, etc.) in India (Kumar et al. 2002; Purohit et al. 2002; Purohit and Fischer 2014). India produces a huge quantity of agricultural and forestry residues/waste, a major part of which is used for domestic, commercial, and industrial activities, viz., fodder for cattle, domestic fuel for cooking, construction material for rural housing, industrial fuel for boilers, manufacturing cardboard, and other similar applications. Biomass constituted more than 85% of India’s rural energy fuel consumption in 2005, the bulk of it being used for meeting cooking energy needs (Chaturvedi et al. 2014). However, the traditional use of biomass have many disadvantages as an energy feedstock primarily due to the low bulk density of traditional biomass feedstocks (Purohit et al. 2006).

To improve the characteristics of traditional biomass feedstocks for transportation, storage, and combustion (e.g., feeding into furnaces), it is necessary to upgrade the raw agricultural and forestry residues by increasing their bulk density through briquetting or pelletization. The pelleting process increases the specific density of biomass to more than 1000 kg m^−3^ (Lehtikangas 2001; Carroll and Finnan 2012; Palšauskas and Petkevičius 2013; Monteiro et al. 2013; Prvulovic et al. 2014). When compared to other types of modern bioenergy, the pellet sector is one of the fastest growing. Apart from increasing volumetric calorific value of raw biomass (Patiño et al. 2008) pelletization also increases the efficiency of thermochemical conversion (Widjaya et al. 2018) due to consistent moisture level (Muth et al. 2014). In the pellet form, non-woody biomass combustion can produce lower ash content compared to combustion of raw heterogeneous material. Holt et al. (2006) reported that the ash product of combusting cotton gin waste pellets was decreased twofold to threefold compared to combusting the unpelleted material. Moreover, biomass briquettes and pellets can also be used as fuel in wood stoves (Pettersson et al. 2011; Roy and Corscadden 2012) and external combustion engines (Cardozo et al. 2014), and as raw material for pyrolysis/gasification (Erlich et al. 2006; Gomez-Barea et al. 2010; Lajili et al. 2018).

The use of biomass pellets in boilers for process heat and power generation applications can be made in two somewhat different ways—(i) boiler can be fired exclusively with biomass pellets or (ii) biomass pellets are co-fired with coal. While the slagging and fouling risks of several biomass feedstocks, given their high alkali, silica, or chlorine contents (Werther et al. 2000; Teixeira et al. 2012; Du et al. 2014) currently limit their application in combustion processes, additives based on different chemical compositions and possible counteracting effects can be used to abate ash-related problems during biomass combustion (Wang et al. 2012; Clery et al. 2018). Ahn and Lee (2014) investigated the potential of non-used forest biomass residues as raw materials for making wood pellets with additives such as wood tar and starch and to evaluate fuel characteristics of the pellets. When the wood tar (10 wt%) was added, the calorific value was increased from 4630 kcal/kg (wood pellet without additive) to 4800 kcal/kg (wood pellet with additive). Moreover, with the increase of additive amount into wood pellet, the length and individual density of wood pellet increased. Recent studies indicate that there is no significant increase in the slagging due to co-firing of biomass along with coal so long as the thermal input contributed by biomass is limited up to 20% (Pedersen et al. 1996; Lu et al. 2008; Wang et al. 2011; Steer et al. 2013) though the optimum proportion of biomass co-firing with coal has been varying for each case of study. Analytical studies using various empirical correlations (McLennan et al. 2000; Pronobis 2005; Yu et al. 2014) suggest that 100% pelletized wood has moderate to high slagging propensity than other fuel combinations. Seepana et al. (2017) discussed about pelletized wood co-firing with high ash^1^ Indian coal by conducting co-milling and co-firing trials in a 1000 kg/h of pilot scale test facility. The probability of slagging may increase while co-firing wood pellets with Indian coal, when compare with 100% coal firing (Seepana et al. 2017).

The different compositions of various biomass species directly affect the pelletizing properties and the ability to form stable pellets. In biomass pelleting manufacture process, several studies have been focused on the mechanisms of process parameters (i.e., pressure, temperature, moisture content, particle size) on pellet quality (Kaliyan and Morey 2009, Carone et al. 2011; Stelte et al. 2012; Jiang et al. 2014). Some of these parameters are related to the raw materials used (Arshadi et al. 2008), whereas others are related to the quality management of the manufacturing process (Gilbert et al. 2009; Stelte et al. 2012). In a simplified model of an ordinary industrial pelletization process presented by Carone et al. (2011), temperature resulted the most important variable influencing pellet mechanical properties, followed by the initial moisture content and the particle size of the raw material. In particular, high process temperature, low moisture contents, and reduced particle sizes allowed obtaining good quality pellets (Carone et al. 2011).

Wood pellets in India are mostly used for residential cooking and heating (with pellet stoves) and/or commercial purposes (Venkataraman et al. 2010; Thurber et al. 2014; Brooks et al. 2016). For example, over 400,000 Oorja stoves (a combination of a uniquely designed “micro-gasification” device or stove and a biomass-based pellet fuel) were sold between 2006 and 2010 in the Indian market (Thurber et al. 2014). In contrast, biomass pellets are being increasingly used for power generation in many countries. In Europe, North America, and Asia (viz., China, Japan, and South Korea), wood pellets are mostly used for co-firing at coal-fired power plants (Baxter 2005; Ehrig and Behrendt 2013; Goh et al. 2013; Johnston and van Kooten 2015; Xian et al. 2015). In India, Section 86(1) (e) of the Electricity Act, 2003 requires the State Electricity Regulatory Commissions (SERCs) to determine and implement Renewable Purchase Obligations (RPOs). To achieve the target set by India’s National Action Plan on Climate Change (NAPCC), the Government of India (GoI) launched the Renewable Energy Certificate (REC) mechanism in November 2010 (Gupta and Purohit 2013). NAPCC aims to derive 15% of India’s energy requirements from renewable energy sources (non-solar) by 2020. India’s Intended Nationally Determined Contributions (INDC), submitted to the United Nations Framework Convention on Climate Change (UNFCCC) before the 21st Conference of the Parties (COP21), also states that it is “envisaged to increase biomass installed capacity to 10 GW by 2022 from the current capacity of 4.4 GW” as part of the overall goal of increasing the share of non-fossil fuel electricity generation capacity to 40% in the country’s electricity mix by 2030 (GoI 2015). Electricity generation through biomass pellets and/or co-firing of biomass pellets with coal can help to meet the RPO targets of SERCs in states with low solar/wind resources (and limited wasteland availability) and with extensive availability of agricultural and forestry residues, or these states can attract investment through REC mechanism.

It is in this context that we will address the following questions here: (a) What is the current and future surplus biomass availability in India? (b) What is the potential for the production of biomass pellets and corresponding generation of electricity in India up to 2030? (c) What is the production cost of biomass pellets? (d) What is the cost of electricity production from BPBP projects? and (e) Is a carbon price required for enhancing the financial viability of BPBP generation?

The paper is set out as follows: the “Methodology: estimating surplus biomass availability, electricity generation potential, and levelized costs” section describes the methodology of the paper. The “Application of the methodology to the case study of India” section presents the key assumptions and input parameters used as an application of the methodology (to the case study of India) for potential assessment of agricultural/forestry residue for biomass pellets. The results are presented in the “Results” section, that is, (i) the estimates of the state-wise availability of agricultural and forestry residues for the production of biomass pellets; (ii) the potential of biomass pellets for electricity generation; and (iii) the techno-economic viability of biomass pellets for electricity generation and the associated carbon finance potential of biomass pellets. The “Discussion” section briefly discusses the potential for employment generation in the biomass pellet-based electricity production value chain, the implications of the climate policy, and the international biomass pellet market. Finally, the “Conclusions and policy implications” section presents the key findings and insights emerging from this study.

## Methodology: estimating surplus biomass availability, electricity generation potential, and levelized costs

The rise in pellet consumption has resulted in a wider variety of materials used for pellet manufacture. Thus, the pellet industry has started looking for a broad range of alternative materials, such as wastes from agricultural activities, forestry, and related industries, along with the combination thereof. In the “Results” section, we present our estimates for the biomass surplus from agriculture, forestry, and wasteland, the potential for biomass pellet-based electricity generation, and its financial viability. This section lays out the methodology for the same.

### Availability of agricultural residue for biomass pellets

Agricultural residues are the most commonly used biomass feedstock for the production of biomass pellets in India. Availability of agricultural residues as energy feedstock essentially depends on the total amount of the crop produced, the residue-to-product (grain) ratio for the crop, the collection efficiency (which includes storage-related considerations), and the amount used in other competing applications. The effective crop residue availability for ith crop (CRA_eff,i_) per unit crop produced can therefore be expressed as (Purohit 2009)

1 $$ {\mathrm{CR}\mathrm{A}}_{\mathrm{eff},\mathrm{i}}={\mathrm{RC}}_{\mathrm{i}}\left(1-{\mathrm{CR}}_{\mathrm{cts},\mathrm{i}}\right)\left(1-{\mathrm{CR}}_{\mathrm{fodder},\mathrm{i}}\right)\left(1-{\mathrm{CR}}_{\mathrm{oth},\mathrm{i}}\right) $$

where RC_i_ represents the residue-to-product (grain) ratio for ith crop; CR_cts_ is the fraction of the total crop residue lost in collection, transportation, storage, etc.; CR_fodder_ is the fraction of the crop residue used for fodder; and CR_oth,_ is the fraction of the crop residue employed in other competing uses.

Therefore, the effective net annual crop residues availability, NRA_eff_, for biomass pellets in India can be estimated as

2 $$ {\mathrm{NRA}}_{\mathrm{eff}}={\sum}_{i=j=1}^{m,n}{A}_{i,j}{Y}_{i,j}{\mathrm{RC}}_i\left(1-{\mathrm{CR}}_{\mathrm{cts},i}\right)\left(1-{\mathrm{CR}}_{\mathrm{fodder},i}\right)\left(1-{\mathrm{CR}}_{\mathrm{oth},i}\right) $$

where *A*_i,j_ and *Y*_i,j_ respectively represent the area and the yield of ith crop (*i* = 1, 2, 3, . . . *m* crop) in the jth state (*j* = 1, 2, 3, . . . *n* state).

For estimating residues from forests and wastelands, a similar approach is followed. Data sources for all variables in Eqs. 1 and 2 are given in “Application of the methodology to the case study of India” section.

### Unit cost of biomass pellets

The unit cost of biomass pellet production, UC_bp_, can be obtained as the ratio of the total annualized cost of the biomass pellet unit to the annual production of biomass pellets. The annual production of biomass pellets, AP_bp,_ can be expressed as:

3 $$ {\mathrm{AP}}_{\mathrm{bp}}=8760{\mathrm{CUF}}_{\mathrm{bp}}{P}_{\mathrm{bp}} $$

where CUF_bp_ represents the capacity utilization factor of the biomass pellet unit and *P*_bp_ is the rated production capacity (kg/h) of the biomass pellet unit.

The total annualized cost would comprise the annualized capital cost, the annual operation cost (including cost of fuel), and the annual repair and maintenance cost. Therefore, the unit cost of biomass pellet, UC_bp_, can be estimated as

4 $$ {\mathrm{UC}}_{\mathrm{bp}}=\frac{\left\{{C}_{\mathrm{bp}}\ R\left(d,{t}_{\mathrm{bp}}\right)\right\}+\left\{{\xi C}_{\mathrm{bp}}\right\}+\left\{8760{\mathrm{CUF}}_{\mathrm{bp}}\left({C}_{\mathrm{l}}{N}_{\mathrm{l}}+{P}_{\mathrm{bp}}{r}_{\mathrm{bf}}{C}_{\mathrm{bf}}+{p}_{\mathrm{e}}{\chi}_{\mathrm{e}}{P}_{\mathrm{bp}}\right)\right\}}{8760{\mathrm{CUF}}_{\mathrm{bp}}{P}_{\mathrm{bp}}} $$

where *C*_bp_ represents the capital investment cost of the biomass pellet unit, *ξ* is the annual repair and maintenance cost as a fraction of the capital cost, *C*_l_ is the cost of the manpower required, *N*_l_ is the number of workers hired, *C*_bf_ is the cost of biomass feedstock, *r*_bf_ is the correction factor for estimating the requirement of biomass feedstock based on the production capacity of the pellet unit (to account for the moisture loss during the drying and pellet production processes), *χ*_e_ is the specific amount of electricity consumption in the biomass pellet unit, *p*_e_ is the unit cost of electricity, and *R* (*d*, *t*_bp_) is the capacity recovery factor which can be estimated as

5 $$ R\left(d,{t}_{\mathrm{bp}}\right)=\frac{d{\left(1+d\right)}^{t_{\mathrm{bp}}}}{\left({\left(1+d\right)}^{t_{\mathrm{bp}}}-1\right)} $$

where *d* is the discount rate and *t*_bp_ is the useful lifetime of the biomass pellet unit.

### Economics of biomass pellet for electricity generation

The levelized cost of electricity is estimated as the ratio of the total annualized cost of the biomass power plant to the annual amount of electricity produced by a biomass power plant using biomass pellets as a feedstock. The annualized cost comprises the annualized capital cost, annual operation cost (including the cost of fuel), and annual repair and maintenance cost.

The net present value (NPV) of a BPBP project can be determined using the following expression:

6 $$ \mathrm{NPV}={\sum}_i^T\frac{B_{\mathrm{i}}-{C}_{\mathrm{i}}}{1+{d}^{\mathrm{i}}}-{C}_{\mathrm{o}} $$

where *T* is the lifetime of the BPBP project. The salvage value of the BPPP project at the end of its useful life has been assumed to be negligibly small in writing the Eq. (6). The net annual monetary benefit accrued to the investor *(B*_i_ *− C*_i_*)* is assumed to be uniform over the useful life of the BPBP project.

## Application of the methodology to the case study of India

Of India’s total geographic area of 328 million hectares (Mha), the net cropped area accounts for approximately 43%, and it appears that the net cropped area has stabilized at approximately 140 Mha since 1970 (CMIE 1997; Ravindranath et al. 2005). The gross cropped area, accounting for the cultivation of multiple crops per year, increased from 132 Mha in 1950–51 to approximately 195 Mha in 2008–09. There are two main cropping seasons in India, viz., Kharif (based on the southwest monsoon) and Rabi (based on the north-east monsoon). The gross cropped area includes land areas subjected to multiple cropping (normally double cropping), mainly in irrigated land. The net irrigated area increased substantially from 21 Mha in 1950–51 to approximately 64 Mha in 2013–14 (MoA 2014). Rice and wheat are the dominant crops, together accounting for 41% of the total cropped area, while pulses, oilseeds, and other commercial crops account for 13.8%, 15.9%, and 10.2% respectively. Cereals dominate the agricultural crops and account for 60% of the total cropped area, followed by pulses, cotton, and sugarcane. The specific ratios of residue-to-grain production of different crops are taken from (Tripathi et al. 1998b; Purohit and Michaelowa 2007; Ravindranath et al. 2011; Purohit and Dhar 2015) and presented in Table 1.

**Table 1:** Area under different crops and their production

Economic produce/crop	Type of residue	Residue-to-grain ratio	Area (Mha)		Crop production (Mt)			
			2010/11	2020/21	2030/31	2010/11	2020/21	2030/31
Food grains								
Rice	Straw + husk	1.8	42.9	48.1	50.3	96.0	109.9	123.2
Wheat	Straw	1.6	29.1	33.7	36.6	87.0	108.2	121.1
Jowar	Stalk	2.0	7.4	5.2	3.4	7.0	6.0	5.7
Bajra	Straw	2.0	9.6	9.3	8.8	10.4	11.4	12.3
Maize	Stalk + cobs	2.5	8.6	8.4	9.0	21.7	24.8	28.3
Other cereals	Stalk	2.0	2.9	2.1	1.5	4.6	3.9	3.8
Gram	Waste	1.6	9.2	8.9	8.7	8.2	8.4	8.6
Tur (arhar)	Shell + waste	2.9	4.4	4.4	4.7	2.9	3.1	3.3
Lentil (masur)	Shell + waste	2.9	1.6	1.7	1.9	0.9	1.2	1.4
Other pulses	Shell + waste	2.9	11.2	12.8	13.2	6.2	6.3	6.8
Oilseeds								
Groundnut	Waste	2.3	5.9	6.0	6.1	8.3	8.9	9.6
Rapeseed and Mustard	Waste	2.0	6.9	7.2	7.9	8.2	9.6	11.0
Other oilseeds	Waste	2.0	14.5	16.7	18.6	16.0	19.3	22.4
Fiber								
Cotton	Seeds + waste	3.5	11.2	11.9	12.6	5.6	6.1	6.4
Jute and Mesta	Waste	1.6	0.9	1.0	1.0	1.9	2.3	2.5
Sugar								
Sugarcane	Bagasse + leaves	0.4	4.9	5.1	5.6	342.4	406.4	459.3
Total			171.0	182.4	190.1	627.3	735.9	825.8

Source: (Ravindranath et al. 2005; Purohit et al. 2006; MoA 2012)

The use of crop residues varies from region to region and depends on the calorific values of individual crops, their lignin content, density, palatability by livestock, and nutritive value. The residues of most cereals and pulses have fodder value. However, the woody nature of the residues of some crops restricts their utilization to fuel use only. The dominant end uses of crop residues in India are as fodder for cattle, fuel for cooking, and thatch material for housing (Purohit and Fischer 2014). India has the largest cattle population of 305 million (Intodia 2017) in the world in 2017 followed by Brazil and China. The straws and stovers of rice, wheat, finger millet, maize, sorghum, bulrush millet, and sugar cane tops are the major lean season feeds used by farmers, alone or supplemented according to availability and the financial status of the farmer (Suttie 2000; Ravindranath et al. 2005). Although India has over 10 Mha of grazing pasture land, grass productivity is low due to climatic conditions and soil degradation, leading to the near-total dependence of cattle on the crop residues of cereals and pulses. The estimated total amount of residues utilized as fodder was 301 Mt in 1996–97 (CMIE 1997) and is estimated at over 363 Mt for 2010–11, accounting for approximately 53% of total residue generation, as shown in Table 2 below (“Results” section). Where cereals are concerned, the use of crop residues as fodder is the top priority in rural areas. Only some rice straw and maize stalks/cobs, as well as ligneous residues are likely to be available for use as an energy source. Moreover, it is assumed that 20% of agricultural residue is lost in the collection, transportation, storage, etc. (Purohit and Dhar 2015).

**Table 2:** Surplus agricultural residue availability for biomass pellets in India

Crop residue	Total residue production(air dry*) – Mt			% of agriculturalresidue used for^**^			Net agricultural residueavailability for biomass pellets^***^		
	2010/11	2020/21	2030/31	Fodder	Fuel	Other	2010/11	2020/21	2030/31
Rice straw and husk	172.8	197.9	221.8	80.8	11.1	8.0	13.8	15.8	17.8
Wheat straw	139.2	173.1	193.7	86.4	0.0	13.6	0.0	0.0	0.0
Jowar stalk	14.1	12.1	11.5	100.0	0.0	0.0	0.0	0.0	0.0
Bajra straw	20.7	22.8	24.7	89.8	0.0	10.2	0.0	0.0	0.0
Maize stalk and cobs	54.3	62.1	70.6	81.0	19.0	0.0	7.4	8.5	9.7
Other cereals stalk	9.1	7.8	7.6	100.0	0.0	0.0	0.0	0.0	0.0
Gram waste	13.2	13.5	13.8	0.0	100.0	0.0	9.5	9.7	10.0
Tur shell and waste	8.3	8.9	9.6	3.5	48.5	48.0	2.9	3.1	3.3
Lentil shell and waste	2.7	3.6	4.1	3.5	48.5	48.0	1.0	1.3	1.4
Other pulses shell/waste	18.0	18.4	19.8	3.5	48.5	48.0	6.3	6.4	6.9
Groundnut waste	19.0	20.6	22.0	0.0	13.2	86.8	1.8	1.9	2.1
Rape and Mustard waste	16.4	19.3	22.1	0.0	100.0	0.0	11.8	13.9	15.9
Other oilseeds waste	32.1	38.6	44.7	0.0	100.0	0.0	23.1	27.8	32.2
Cotton seeds and waste	19.6	21.2	22.5	0.0	100.0	0.0	14.1	15.3	16.2
Cotton gin trash	0.4	0.5	0.5	0.0	100.0	0.0	0.3	0.3	0.4
Jute and Mesta waste	3.1	3.6	3.9	0.0	100.0	0.0	2.2	2.6	2.8
Sugarcane bagasse/leaves	137.0	162.6	183.7	11.8	41.0	47.2	28.7	34.1	38.6
Total	679.9	786.3	876.6				123.0	140.8	157.2

^*^Moisture content (At harvest: 30%; at use: 10%)

^**^Source: (Ravindranath et al. 2005; Purohit 2009)

^***^Apart from fodder and other applications, the net agricultural residue availability for biomass pellets also takes into account the residue used for biomass power/cogeneration projects

**Fig. 2:**
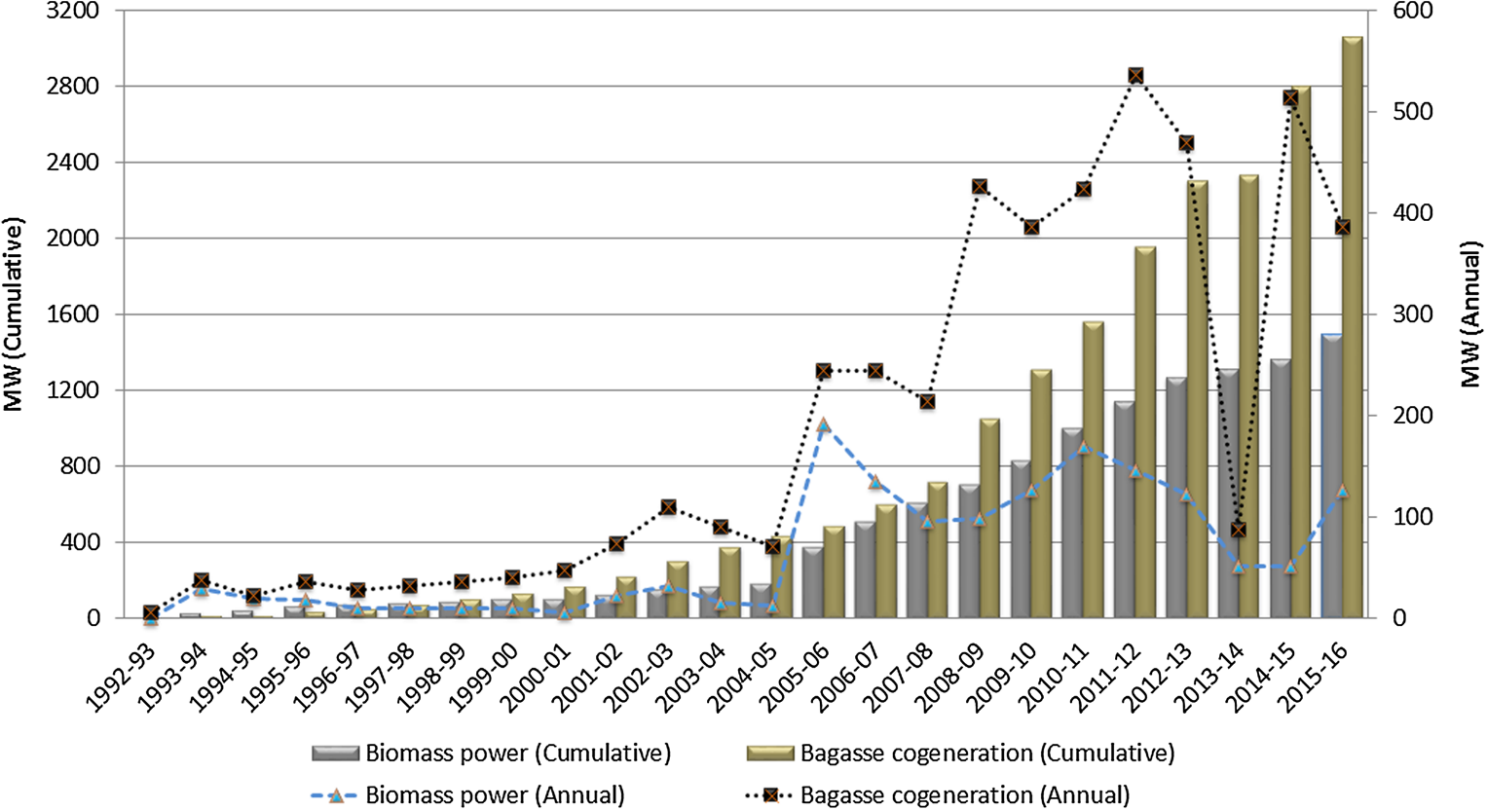
Installed capacity of biomass power/cogeneration projects in India (source: MNRE annual reports)

Another major alternative application of non-fodder and non-fertilizer agricultural residues is biomass power and bagasse cogeneration. India’s Ministry of New and Renewable Energy (MNRE) implemented the Biomass Power/Cogeneration program with the main objective of promoting technologies for the optimum use of the country’s biomass resources for grid power generation. The program is encouraged through a conducive policy at the state and central levels. Figure 2 presents the installed capacity of biomass power/cogeneration projects in India until December 2016. As of April 2018, 8701 MW grid-interactive biomass/bagasse cogeneration power projects had been installed, and an additional 675 MW of biomass (non-bagasse) cogeneration and 163 MW of biomass gasification off-grid projects have also been installed in India (MNRE 2018). Therefore, the use of a significant proportion of agricultural residues for power generation has to be accounted for when estimating the net biomass pellet potential from agricultural residues. For the base year 2010–11, the installed capacity of grid-connected bagasse cogeneration projects was 1562 MW (MNRE 2011). Using a specific bagasse consumption level of 1.6 kg/kWh (Purohit and Michaelowa 2007) and a capacity utilization factor (CUF) of 53% (MNRE 2013), the amount of bagasse used in the cogeneration projects is estimated at 11.6 Mt, which is 20% of the bagasse availability for energy applications (Brooks et al. 2016). Similarly, the cumulative installed capacity of grid and off-grid biomass power/cogeneration projects was 1400 MW^2^ (MNRE 2011). Using the specific biomass consumption level of 1.21 kg/kWh (Purohit 2009) and CUF of 80% (MNRE 2012), the biomass used in the power/cogeneration projects is estimated at 11.8 Mt, which is approximately 10% of (non-bagasse) agricultural residues available for energy applications. This share of residues used for power/cogeneration is kept constant in the estimation of the net biomass pellet production from agricultural residues in the near future.

## Results

### Biomass surplus for biomass pellets in India

#### Biomass surplus from agricultural residues

**Fig. 3:**
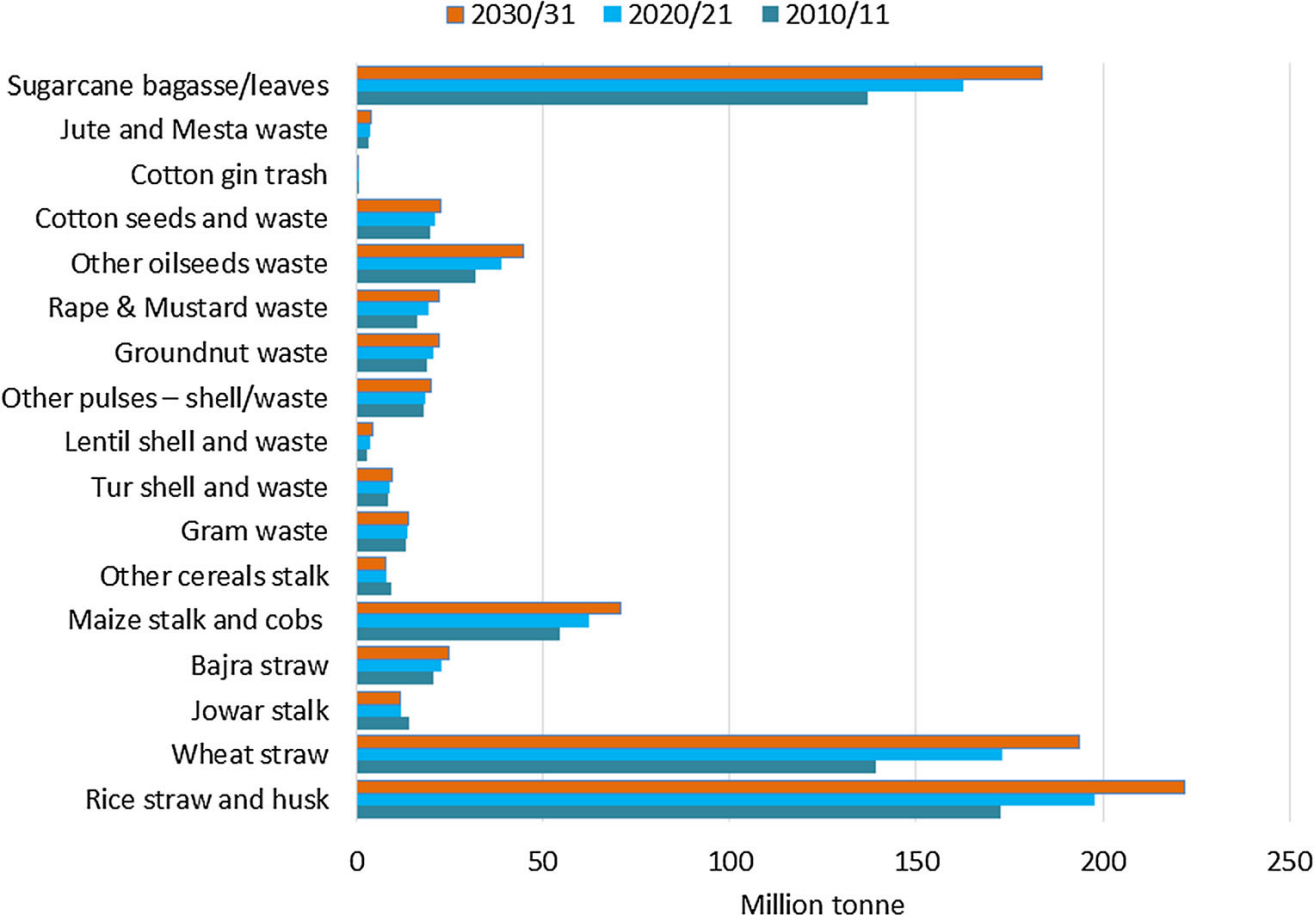
Gross residue availability from crop production in India. Moisture content (air day): 30% for bagasse and 10% for all other agri-residues (source: own estimates)

Table 1 presents the area under cultivation and the production of different crops (MoA 2012). For 2020–21 and 2030–31, the area and crop productivity were projected based on the data from 1950–51 to 2011–12 (Figs. S1–S6) as shown in Section S1 of the supplementary section. Figure 3 presents the total residue production in India based on the production of different food grains, oilseeds, fibers, and sugarcane. For 2010–11, the area under cultivation and total crop production were 171 Mha and 627 Mt respectively. The gross residue availability is estimated at 680 Mt for 2010–11. Hiloidhari et al. (2014) reported a gross crop residue production of 686 Mt during 2010–11 by considering 39 residues from 26 crops as compared to the 16 principal crops examined in this study. Singh and Gu (2010) reported a gross potential of 1055 Mt/year, including residues from spices (ginger, cardamom, coriander, garlic, cumin, and dry chili) and plantation crops (such as rubber and coffee), while the present study and Hiloidhari et al. (2014) did not include these residues. The highest average densities of agricultural residues of more than 500 tonnes/km^2^ were observed for Punjab and Haryana, where intensive wheat–rice systems are practiced on mostly irrigated land (Purohit and Dhar 2018). For 2010–11, agricultural residue availability for energy applications is estimated at approximately 150 Mt in 2010–11^3^ with a collection efficiency of 80% (Purohit and Dhar 2015). In the base year, agricultural residue availability from select crops for biomass pellets is estimated at 123 Mt after adjusting moisture content for bagasse and residue used for biomass/bagasse-based power generation as shown in Table 2. The net residue availability for biomass pellets in 2020/21 and 2030/31 is estimated at 141 Mt and 157 Mt respectively. In our estimation, this potential for biomass pellet production represents approximately 20% of the theoretical maximum obtainable if all crop residues (e.g., straw, husks, stalks, cobs, shells, bagasse, etc.) were to be converted into biomass pellets (Table 2).

#### Biomass surplus from forestry/wasteland

**Fig. 4:**
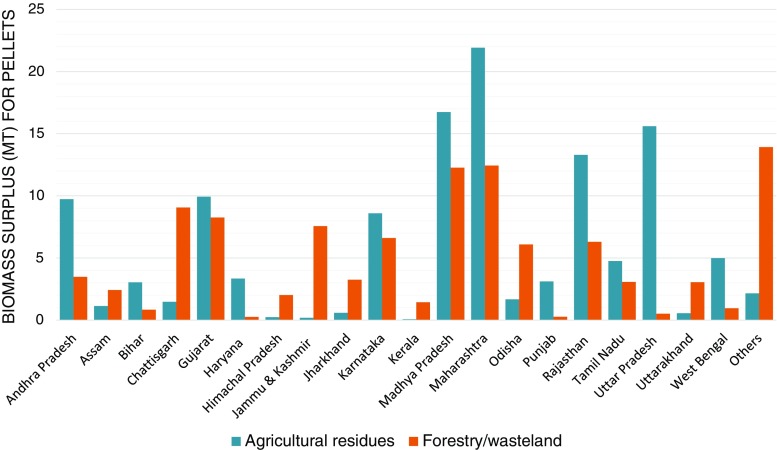
State-wise biomass surplus for biomass pellets in India (source: IISc (2015) and own estimates)

India witnessed a 24% rise in forest cover between 1950 and 2010, increasing from around 40.48 to 68 Mha during this period (FAO 2011; MoEF 2012). The 2013 Forest Survey of India states that the forest cover increased to 69.8 Mha by 2012, as per satellite measurements; this represents an increase of 5871 km^2^ of forest cover in 2 years (MoEF 2014). For 2002–04, the total surplus biomass from forest and wastelands is estimated at 104 Mt (IISc 2015) as per the Biomass Atlas of India (Table 3). The total surplus biomass from forest and wastelands will increase in the near future due to the increase in forest cover. However, a significant amount of forest residues is consumed by the population residing in or near the forest, and the plantation products are used by the timber, paper, and pulp industries. Therefore, we have kept the biomass surplus from forest and wastelands as constant at the 2004 level. Figure 4 presents the biomass surplus available from agriculture and forestry/wasteland for the production of biomass pellets in India. The northern states such as Punjab, Haryana, and Uttar Pradesh have large amounts of agricultural residues available for biomass pellets as compared to the biomass surplus from forestry/wasteland. It may be noted that the hilly states in the north (J&K, Himachal Pradesh, and Uttarakhand) and the north-east have a large amount of surplus biomass from forestry/wasteland as compared to the agricultural sector.

**Table 3:** State-wise biomass surplus through forestry and wasteland

State	Area(Mha)	Gross availability of biomassfrom forestry/wasteland (Mt)	Net availability of biomassfrom forestry/wasteland (Mt)
Andhra Pradesh	3.6	5.2	3.5
Arunachal Pradesh	5.5	8.3	6.0
Assam	2.7	3.7	2.4
Bihar	0.9	1.2	0.8
Chhattisgarh	8.8	13.6	9.1
Goa	0.2	0.2	0.1
Gujarat	9.0	12.2	8.3
Haryana	0.3	0.4	0.3
Himachal Pradesh	2.3	3.1	2.0
Jammu and Kashmir	9.8	11.5	7.6
Jharkhand	3.5	4.9	3.2
Karnataka	7.0	10.0	6.6
Kerala	1.2	2.1	1.4
Madhya Pradesh	12.8	18.4	12.3
Maharashtra	13.2	18.4	12.4
Manipur	1.3	1.3	0.8
Meghalaya	1.5	1.7	1.1
Mizoram	1.6	1.6	1.1
Nagaland	0.8	0.8	0.6
Orissa	6.3	9.4	6.1
Punjab	0.2	0.4	0.3
Rajasthan	14.1	9.5	6.3
Sikkim	0.4	0.5	0.4
Tamil Nadu	3.2	4.7	3.1
Tripura	0.8	1.0	0.7
Uttar Pradesh	3.9	5.5	3.7
Uttaranchal	2.9	4.6	3.1
West Bengal	1.1	1.4	0.9
All India	118.8	155.5	104.0

Source: IISc (2015) and own estimates

### India’s electricity generation and potential of biomass pellets

**Fig. 5:**
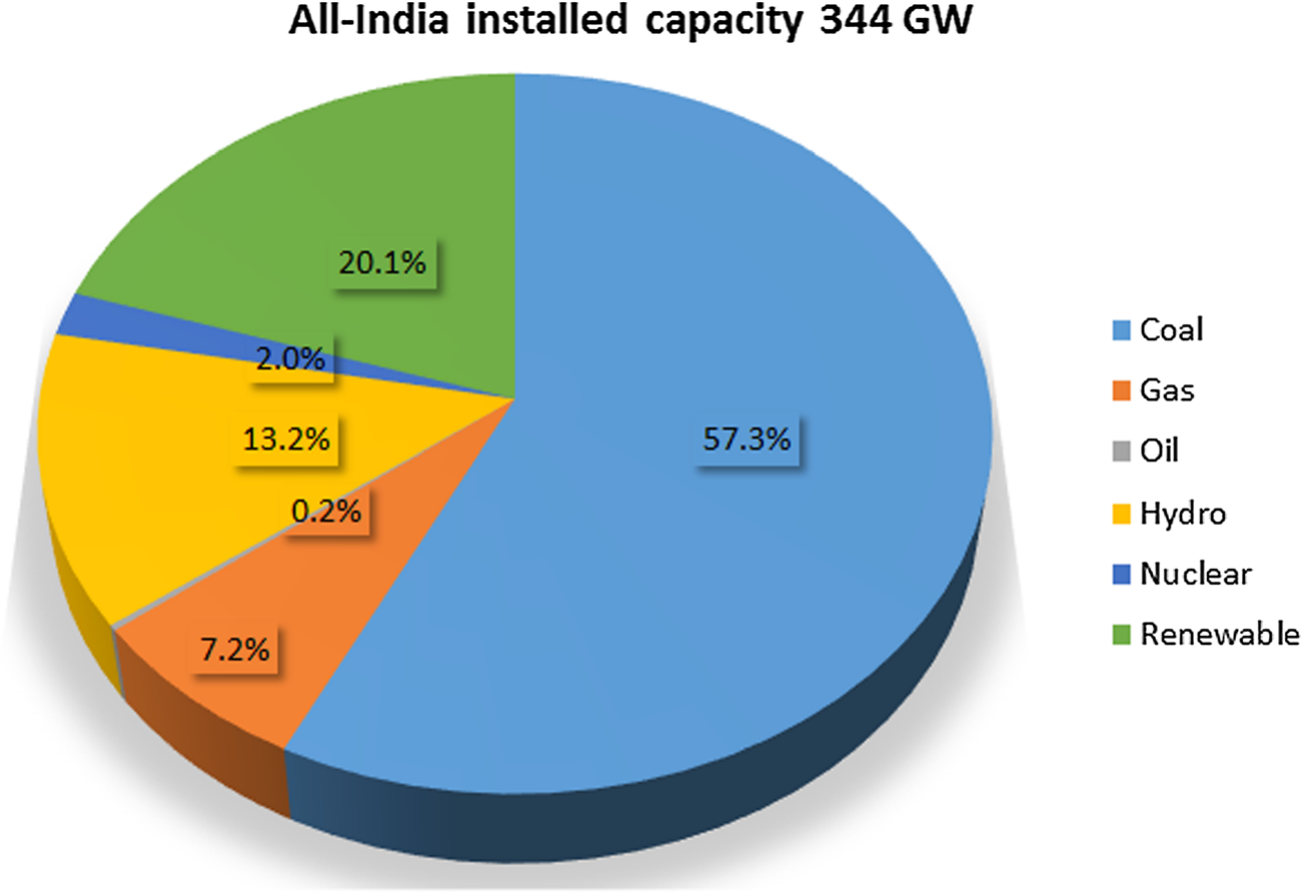
Installed capacity of power plants in India until March 2018 (source: CEA 2018)

The utility electricity sector in India had an installed capacity of 344 GW as of 31st March 2018 (CEA 2018). The share of renewable (including large hydro) power plants constituted 33.2% of the total installed capacity, and non-renewable power plants constituted the remaining 66.8% (Fig. 5). As coal is the mainstay of India’s electricity production and is expected to continue to remain so in the near future as well, it is important to understand the role of coal in India’s power generation. The installed capacity of coal thermal power plants was 197.2 GW in March 2017, that is, around 57% of the total installed capacity in the country (CEA 2018). Sub-critical pulverized coal (PC) technology is currently used in most of the coal-based thermal power plants in the country. All newly constructed coal thermal power plants in India are expected to be based on super-critical technology. Coal is required in large quantities for power generation, and India has abundant reserves of this fossil fuel. Coal consumption for power generation increased from 278 Mt in 2004–05 to 546 Mt in 2015–16 (CEA 2017) and is expected to increase to over 1000 Mt in 2030–31 (IEA 2014), using World Energy Outlook (WEO) current policy scenario trends provided by the International Energy Agency (IEA). India’s proven non-coking coal resources, used primarily for power generation, are about 100 billion tonnes (MoC 2013). However, indigenous coal production has not been able to meet domestic demand, and hence a significant proportion of coal is imported. Currently, about 25% of India’s coal supplies are imported (Rathnam et al. 2013). Whether this number will change in the future depends on the rate at which domestic production grows, as well as the movement of coal prices in the international market. Domestic coal production is increasing primarily due to the domestic reforms in the coal sector, and international coal prices have also increased significantly in the last 2 years. Based on these two factors, it appears that Indian coal imports in the future will not increase in terms of share of total coal consumption. In the long run, if the cost of biomass pellet-based electricity generation becomes competitive with the cost of coal production, a proportion of the coal imports could be replaced by biomass imports as well. As shown in the “Application of the methodology to the case study of India” section, the biomass pellet trade is increasing. Stringent climate policy requirements could further compel India to start seriously thinking about biomass pellet imports.

**Fig. 6:**
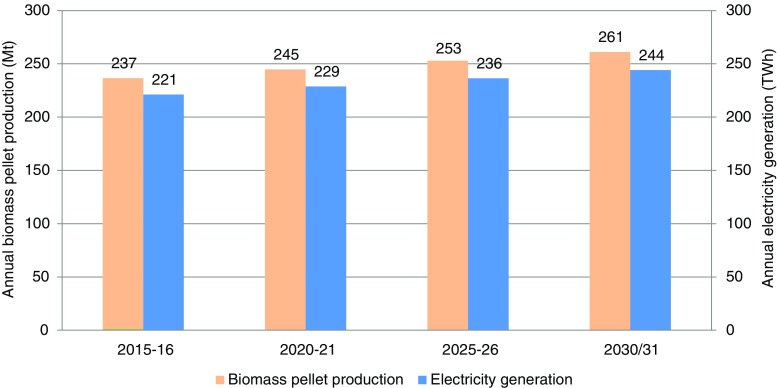
Annual biomass pellet production through biomass surplus and associated electricity generation (source: own estimates)

The annual gross and net availability of agricultural and forestry/wasteland residues and/or waste have been estimated in the previous section. The net biomass surplus availability from agricultural residues and forestry/wasteland for biomass pellet production is estimated at 227 Mt for 2010–11; it is expected to increase to 245 Mt in 2020–21 and to 261 Mt in 2030–31. The surplus^4^ biomass availability from the agriculture sector (133 Mt in 2015–16) alone is sufficient to substitute approximately 25% of the current coal consumption of 531 Mt (CEA 2015) in the power sector (through the co-firing of coal with biomass pellets). Figure 6 presents the annual biomass pellet production based on surplus biomass available from agricultural residues and forestry/wasteland. The associated electricity generation potential of BPBP projects is estimated at 229 TWh for 2020–21 and is predicted to increase to 244 TWh in 2030–31. The associated CO_2_ mitigation potential available through the substitution of coal is estimated at 192 and 205 Mt CO_2_eq in 2020–21 and 2030–31 respectively using the baseline of 0.82 kg CO_2_e/kWh (CEA 2014) if the entire biomass surplus available from the agriculture and forestry/wasteland sectors were diverted for power generation. Apart from CO_2_ reduction from coal substitution use of biomass pellets in India can help in productive use of agricultural/forestry residues. Farmers have traditionally burned excess residues as a means of quick disposal. An estimated 7–8 million tonnes of rice residue associated with post-monsoon agricultural burning are burned each year in Punjab, India (Kumar and Joshi 2013). Cusworth et al. (2018) demonstrated that in October and November, a peak burning season in nearby Punjab about half of all pollution in Delhi can be attributed to agricultural fires on some days. Utilization of surplus residues for power generation potentially reduces both air pollution and GHG emissions.

### Techno-economic evaluation of electricity generation through pellets

The current market price of biomass pellets in India is €0.17 to €0.21/kg (Jain et al. 2015). These pellets are mainly used for cooking application in the commercial sector. To ensure the success of a BPBP project, the biomass pellet unit should be installed near the project. The following sections discuss the cost of biomass pellets, the levelized cost of electricity generation through biomass pellets, the indicators of economic performance, and the impact of the internalization of secondary benefits such as the beneficial effects of CO_2_ emission mitigation on the financial/economic feasibility of a BPBP plant.

#### Cost of biomass pellets

**Fig. 7:**
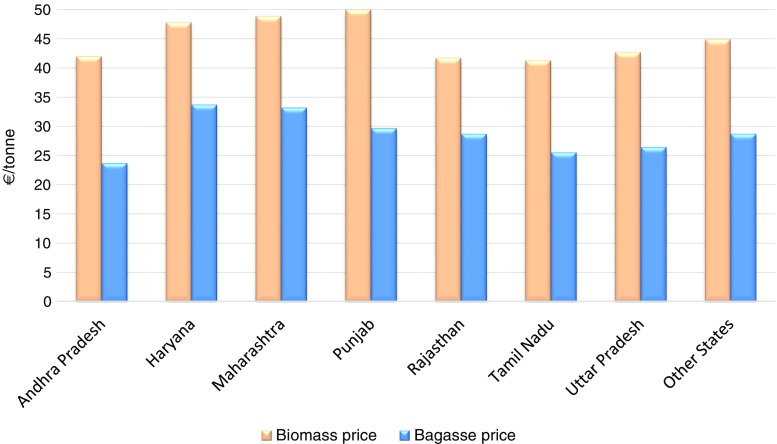
Biomass and bagasse price (€/tonne) by state in India (source: CERC 2015)

The Central Electricity Regulatory Commission (CERC) of India, in terms of Regulation 44 of the Renewable Energy Tariff Regulations, had specified the biomass fuel price applicable during 2012–13 (CERC 2015) and had also specified the fuel price indexation mechanism, in case the developer wished to opt for it, for the remaining years of the control period. Figure 7 presents the biomass and bagasse price applicable for FY 2015–16 by the states. The cost of biomass pellet units and other technical details (Table 4) have been obtained from Nishant Bioenergy Ltd., Mohali, Punjab. The unit cost of biomass (non-bagasse) is taken to be €50/tonne for Punjab. The total electricity consumption for biomass pellet production essentially depends on the type of biomass feedstock (fine or coarse granular/stalky) being used and the moisture content of the biomass feedstock. Drying consumes energy in the form of heat, while size reduction, densification, and cooling operations require electric power input. Mani et al. (2006) observed that the drying process consumes more than 80% of energy, which results in the high energy cost of the pelleting operation. Energy demand for wood pelleting (including all stages, from the reception of raw material to packing) is generally in the range of 80 to 150 kWh/tonne for electricity and around 950 kWh of heat/tonne of water to be vaporized (EBIA 2012). In this study, we have considered specific electricity consumption of 100 kWh/tonne assuming that the biomass feedstock is air dried (moisture content 10%).

**Table 4:** Technical and economic parameters used in the unit cost of biomass pellets

Parameter	Symbol	Unit	Value
Capacity of biomass pellet unit	*P*_bp_	kg/h	1500.00
Capital cost of biomass pellet unit	*C*_bp_	€*	28,571.00
Discount rate	*d*	%	10.00
Useful lifetime of biomass pellet unit	*t*_bp_	Years	10.00
Repair and maintenance cost of biomass pellet unit as a fraction of capital cost	*ξ*	%	10.00
CUF of biomass pellet unit	CUF_bp_	%	68.00
Cost of manpower	*C*_l_	€/man-h	0.57
Number of manpower	*N*_l_	Number	5.00
Cost of biomass feedstock	*C*_bf_	€/kg	0.05
Specific electricity consumption in biomass pellet unit	*χ*_e_	kWh/kg	0.10
Unit cost of electricity	*p*_e_	€/kWh	0.09

Source: As per telephonic interview with a representative of Nishant Bioenergy Ltd., Mohali, Punjab (http://www.nishantbioenergy.com/)

*1 Euro = INR 70/-

**Fig. 8:**
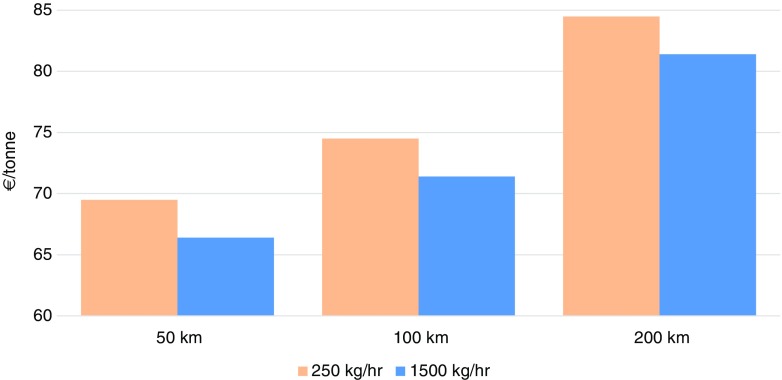
Unit cost of biomass pellet (€/tonne) at different biomass feedstock transportation distances (source: CERC 2015)

Table 4 presents the techno-economics parameters used in the estimation of the cost of biomass pellet production. The biomass pellet unit works 20 h/day and 300 days in a year as per Nishant Bioenergy Ltd. of Mohali, Punjab. Using Eq. (3) to Eq. (5), and based on the key assumptions and input parameters given in Table 4, the unit cost of pellet production is estimated at €64 per tonne for a 1500 kg/h biomass pellet unit (Fig. 8). For a small unit with a rated capacity of 250 kg/h, the unit cost of pellet production is estimated at a higher figure, €67 per/tonne, due to economies of scale.An average agricultural residue transportation cost of € 2.5/tonne for a distance of 50 km is also incorporated in the unit cost of biomass pellet using the methodology developed by Tripathi et al. (1998b).

Figure 8 shows how the unit cost of biomass pellets can increase depending on the transportation distance. An increase in the transportation distance from 50 to 100 km leads to a 4% increase in the unit cost of pellets. The unit cost will further increase with the high moisture content of the biomass feedstock, the type of biomass feedstock used (cutting of stalky materials), and the long transportation distance from the farm gate to the biomass pellet unit, as shown in Fig. 8.

**Fig. 9:**
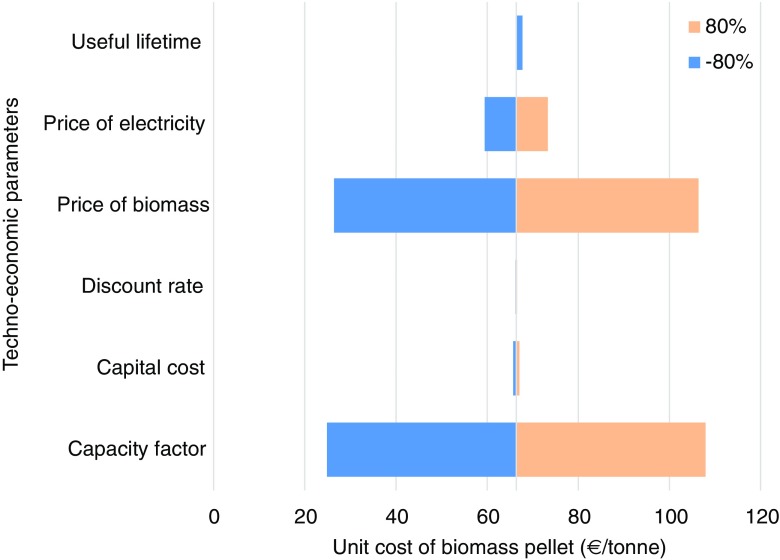
Sensitivity analysis for the unit cost of *C*_bp_ biomass pellet w.r.t. capital cost, CUF capacity utilization factor, *p*_b_ biomass feedstock cost, *p*_e_ price of electricity, *d* discount rate, *t*_bp_ useful lifetime of the biomass pellet unit (source: own estimates)

Figure 9 shows the results of a sensitivity analysis of the effect of uncertainties associated with some important input parameters used in the analysis of the unit cost of biomass pellets. The unit cost of biomass pellets is found to be highly sensitive to the price of biomass feedstock and the CUF of the biomass pellet unit, followed by the price of electricity. It is observed that the capital cost, the discount rate, and the useful lifetime of the biomass pellet unit have a rather moderate effect on the unit cost of pellet production.

#### Levelized cost of electricity generation through biomass pellets

The levelized cost of electricity produced through biomass pellets can be obtained as the ratio of the total annualized cost of the biomass power plant to the annualized cost of electricity generation. CERC considered the capital expenditure (capex) for independent biomass projects as €0.87 million/MW and the operation and maintenance (O&M) cost for a 10 MW biomass power as ~ €0.1 million/MW (CERC 2015). Biomass-fired boilers fueled mainly on paddy straw/Juliflora require a lot of mechanization for collection and pre-processing, for which the developer has to make additional investment to procure equipment like tractors, trolleys, rippers, dozers, and balers. Use of biomass pellets essentially reduces the cost of the fuel supply chain mechanism due to the uniform size of pellets. The cost of biomass pellets is estimated at €64 per tonne as per the estimates presented in the previous section.

**Fig. 10:**
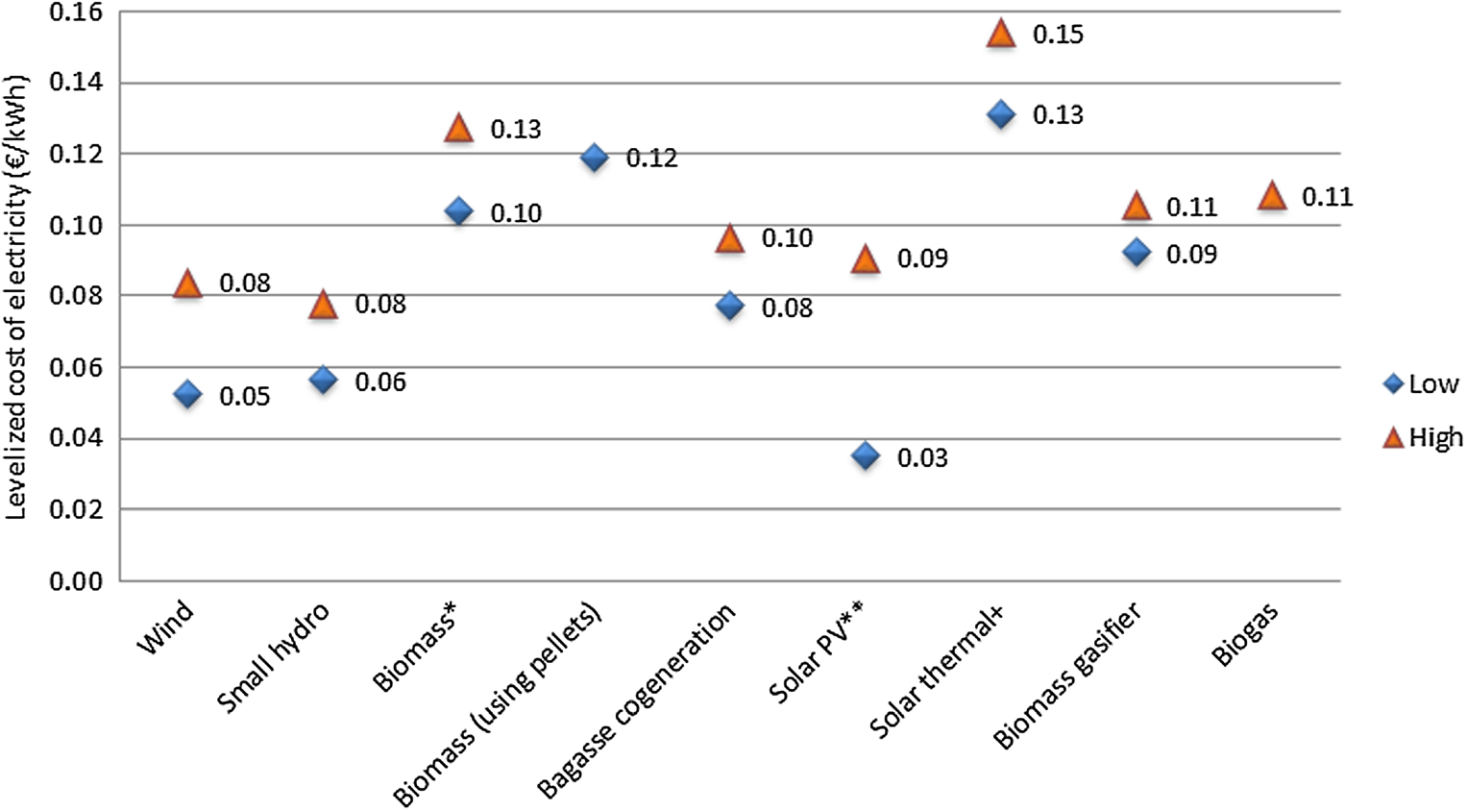
Levelized cost of electricity for biomass power plant (using biomass pellets) along with generic tariff for renewable energy technologies for FY 2015–16 (lower and upper values of the levelized cost of electricity through wind is for different wind zones (CUF = 20% for zone 1 and CUF = 32% for zone 5); lower and upper values for small hydro projects are different for Uttarakhand, Himachal Pradesh, the North-eastern states, and other states (CERC 2015)). ^*^Biomass power projects (other than rice straw- and Juliflora (plantation)-based project). ^**^Lowest solar PV tariff of INR 2.44 (€0.03)/kWh for the 500 MW Bhadla Phase-III Solar Park by Acme Solar. ^+^Lowest solar thermal tariff of INR 9.2 (€0.13)/kWh for parabolic trough systems. Source: (CERC 2015; MNRE 2017; Purohit and Purohit 2017)

Transportation of biomass pellets from the biomass pelletization unit to the BPBP project tacks on additional transportation cost. In this study, the biomass pellet units are assumed to be located within a radius of 50 km of the plant. Therefore, the cost of biomass pellets at the BPBP project will be €66.4 per tonne after including the transportation cost. The useful lifetime of the 100% BPBP plant is taken to be 20 years, and an auxiliary consumption of 10% is considered to assess the levelized cost of electricity (CERC 2015). The levelized cost of BPBP projects is estimated at €0.12/kWh, which is higher than the levelized cost of imported coal-based power production at €0.10/kWh (Shrimali et al. 2015). Figure 10 presents the levelized cost of electricity from biomass power plants using biomass pellets along with the net levelized tariff (after adjusting for accelerated depreciation benefit, if availed) for renewable energy technologies for FY 2015–16 (CERC 2015). The net levelized tariff for biomass power projects (other than rice straw- and Juliflora (plantation)-based projects) with air-cooled condenser and traveling grate boiler is higher than the levelized cost of BPBP projects in the major states of India.

In India, the current electricity tariffs across states/provinces do not reflect the actual cost of supply to different consumer groups. Industrial and commercial consumers, particularly high-voltage consumers, are charged substantially more than the cost of supply, whereas the agricultural sector and, to a lesser extent, the residential sector, are heavily subsidized. The CERC tariff for biomass power projects vary across the states depending on the availability of biomass feedstock, the price of biomass feedstock, the technology used for the condenser (water-cooled or air-cooled) and boiler, etc. A lower tariff of €0.1/kWh for Tamil Nadu is provided for biomass power projects with water-cooled condenser and traveling grate boiler, whereas a higher tariff of €0.12/kWh for Punjab is provided for biomass power projects with an air-cooled condenser and traveling grate boiler (Fig. 10). It may be noted that the above-mentioned tariff rates are not applicable to rice straw- and Juliflora (plantation)-based projects. We have used the lower tariff of €0.1/kWh for biomass power projects provided by CERC (2015) for FY 2015–16 for which the NPV of the BPBP project is negative. Internalization of secondary benefits such as emissions trading improves the financial feasibility of BPBP projects for which the break-even price of carbon is estimated at €18 per tonne CO_2_ (assuming 1 € = INR 70).

## Discussion

### Creation of rural jobs

The large-scale use of biomass pellets for power generation could play an important role in stimulating the local economy and in hastening industrial development. Job opportunities in the biomass pellet development sector will mainly come from the collection, transportation, and processing of biomass feedstock (agricultural/forestry residues) and from the manufacturing of biomass pellets. As per our telephonic conversation with a representative of Nishant Bioenergy Ltd. of Mohali, Punjab, five persons are employed full time for the biomass pellet unit with a capacity of 1500 kg/h (9,000 tonnes/year). In addition, for the transportation of agricultural residues from the farm/industry site to the biomass pellet unit, it is assumed that a truck of 6 tonnes carrying capacity is used three times a day to transport the raw material within a 50-km collection radius. To meet the biomass feedstock requirement on a daily basis, two trucks are needed, with a driver and a loader assigned to each truck. Our preliminary estimates indicate that the biomass pellet production process could generate 224,000 full-time employments in biomass pelletization and in the transportation of agricultural and forestry residues if the entire biomass surplus were diverted to the biomass pellets route. Moreover, the collection and storage of biomass and the manufacturing of biomass pellets are estimated to also create indirect jobs. For 2030/31, it is expected that the biomass pellet industry will create over 260,000 full-time employments in rural areas.

Additional employment will be generated during the construction of biomass power plants. A 10 MW biomass power project can generate employment for approximately 100 workers during the 18-month construction phase, 25 full-time workers for the operation of the facility, and 35 workers for the collection, processing, and transportation of biomass material, as per MNRE estimates (MNRE 2015). With a CUF of 80% and a specific biomass pellet consumption of 1.15 kg/kWh, the related potential of biomass power plants (using pellets) is estimated at 30 GW for 2010–11 and at approximately 35 GW for 2030–31. This translates to over 6.3 million man-months in the construction of 35 GW biomass power plants and 87,500 full-time employments in the operation of biomass power plants for 2030–31.

### Climate policy and the international biomass pellet market

Trade in biomass pellets has already started and has been driven to a large extent by climate policy concerns. EU political and regulatory policy interventions have incentivized wood pellets as a vehicle to help de-carbonize the energy sector. More than 190 countries have submitted their INDCs to the UNFCCC, and these are reflected in the Paris Agreement at the 21st Conference of the Parties (COP-21). Preliminary analysis has shown that the INDCs are a step forward in the direction of global GHG mitigation, but the efforts are still far from what is required to align with a 2 °C pathway.^5^ The pathway chosen by the world till 2030 implies that emission mitigation will have to be even deeper post 2030. Negative-emission technologies like biomass with carbon capture and storage have been proposed as an important part of the technology suite under the 2 °C pathway, although these technologies have yet to be commercialized. Nevertheless, this implies that biomass is being adopted on a large-scale in the shape of dedicated commercial plantations across the world. Not all countries, however, will be biomass suppliers. Land-use modeling within integrated assessment models shows that countries in Latin America will be net exporters of biomass, while many other regions of the world will be producers of bioenergy (Chaturvedi et al. 2015). A stringent climate-control policy will fundamentally change the architecture of the international energy trade. India is not expected to be a significant producer of dedicated bioenergy in the future, not just because of land constraints, but also because of water stress. Thus, if Indian energy systems also need to shift towards bioenergy in a big way, the country will have to become an importer of bioenergy. India has significant other resources like solar energy, but these will not lead to negative emissions, which will be required if the INDC emission pathway till 2030 has to shift towards 2 °C as a long-term goal. Hence, it is important to invest in developing domestic bioenergy resources as much as possible to minimize potential imports, as well as move India towards a low-carbon economy.

### Pelletized versus non-pelletized biomass

**Fig. 11:**
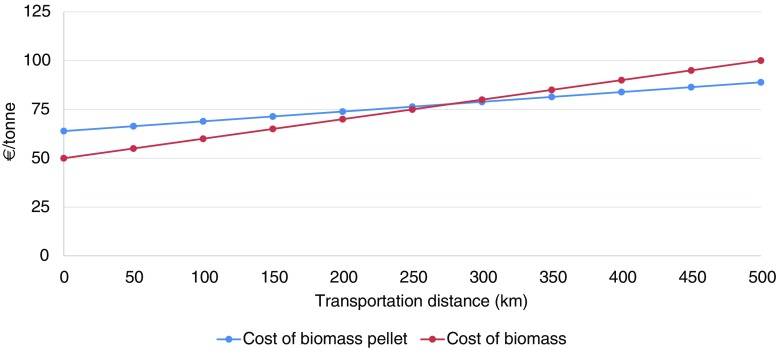
Price of biomass pellet and unprocessed biomass feedstock at different transportation distance (source: own estimates)

Our cost analysis shows that the cost of biomass pellet-based electricity production is well within the range of tariff provided by CERC. However, there is one critical variable that could completely change the economics of biomass-based electricity—the transportation cost of biomass. Figure 11 presents the price of biomass pellets and unprocessed biomass feedstock at different transportation distances. It may be noted that at a critical distance of approximately 275 km, the cost of biomass pellets will be similar to the cost of unprocessed biomass feedstock, whereas, at distances of more than 275 km, the cost of biomass pellets will be lower than the cost of unprocessed biomass feedstock. The market price of biomass feedstock is taken to be €50 per tonne (Table 4) and the cost of biomass pellets is estimated at €64 per tonne. The carrying capacity of the truck is assumed to be 6 tonnes of biomass pellets and 3 tonnes of unprocessed biomass. The pelletization process can increase the bulk density of biomass from an initial bulk density of 40–200 kg/m^3^ to a final compact density of 600–1200 kg/m^3^ and significantly reduce the transportation cost (Mani et al. 2003; Adapa et al. 2007). For this reason, it is, generally, only economically feasible to transport unprocessed biomass for short distances. However, if there is a geographical region that has limited biomass residue and has to still increase the penetration of biomass-based electricity, it makes economic sense to invest only in BPBPs.

The pelletization process resolves some typical problems of biomass fuels: transport and storage costs are minimized, handling is improved, and the volumetric calorific value is increased. Pelletization may not increase the energy density on a mass basis, but it can increase the energy content of the fuel on a volume basis. Hence, for long-distance transport, it makes sense to transport pellets rather than biomass feedstock only. Our analysis also shows that the potential of the different states varies in terms of agriculture and forestry residues. For example, Tamil Nadu has limited potential for producing forest and agriculture residue. The adjoining states of Karnataka and Andhra Pradesh, in contrast, have significant potential. Tamil Nadu’s population exceeds Karnataka’s population by 20% and Andhra Pradesh’s population by 40%. For increasing domestic electricity generation capacity based on biomass, it makes sense for Tamil Nadu to import biomass pellets, as compared to biomass feedstock, from either Andhra Pradesh or Karnataka. This will significantly reduce the cost of feedstock. The same is true for Kerala as well. The states thus have to decide whether to invest in the production of biomass pellets based on their own potential for residue generation, or the biomass-based electricity generation target they want to achieve, or the excess residue potential in the adjoining states.

Based on the financial analysis undertaken by us, we conclude that the cost of electricity production based on the import of biomass pellets from other states will be higher. This scenario will only be possible if there is a high carbon price or if there are stringent targets for biomass-based electricity generation for states that do not have surplus agricultural/forestry residue availability.

## Conclusions and policy implications

Modern bioenergy is being recognized as an increasingly important low-carbon resource by policy-makers around the world to meet climate policy targets. In India also, there is a clear recognition of the significant role of bioenergy in electricity generation as well as in other applications. Bioenergy for power generation can be used in two different forms—pelletized and non-pelletized. The non-pelletized form has been used for a long time for co-firing in coal thermal power plants or biomass power plants. Biomass pellets are now being used extensively and international trade is increasing year on year, largely driven by climate policy targets adopted by developed countries. We focus on this form of energy and estimate the potential for the use of biomass pellet production in India, and the potential for electricity generation from it. We then estimate the cost of 100% biomass pellet-based electricity production and assess its financial viability.

After allocating biomass feedstock for key existing uses of agriculture and forest residues, including for fodder and other competing uses, we estimate that the net residue availability for biomass pellet production will increase from 227 Mt in 2010/11 to 281 Mt in 2030/31. The surplus biomass availability from the agricultural sector (123 Mt in 2010–11) alone was sufficient to substitute 25% of current coal consumption in the power sector (through the co-firing of coal with biomass pellets). The annual electricity generation potential from biomass pellets is estimated to be 244 TWh in 2030/31 out of a total 4000 TWh of electricity production in India in 2030/31. Thus, pelletized biomass can potentially produce 6% of India’s total electricity in 2030/31, in addition to direct biomass co-firing for electricity production.

The cost of biomass pellet-based electricity generation will ultimately determine whether its technical potential will be utilized or not. Based on detailed assumptions concerning input cost and technical factors, we estimate that the cost of a biomass pellet would be €64 per tonne. The levelized cost of electricity will be €0.12 per kWh, which is even higher than the cost of imported coal-based electricity. However, this is within the range of the non-pelletized biomass-based generic levelized tariff as determined by CERC.

We find that, generally speaking, the financial viability of biomass pellet-based electricity generation depends on the surplus availability of biomass feedstock, the price of biomass, the transportation distance from the farm to the BPBP project, and the cooling technology, which together determine the tariff provided by CERC. A carbon price can play an important role in increasing the penetration of biomass pellets in India’s electricity generation if the tariff is at the lower end of the range as determined by CERC. For states with lower tariff for biomass power, the break-even price of carbon for BPBP projects is estimated at 18 Euros/tCO_2_.

India’s status as a primarily agrarian economy makes it the perfect candidate for a bioenergy-led model of energy generation and sustainable development, utilizing the country’s large volumes of leftover agricultural and forestry residues as well as civic waste, and generating income and employment opportunities, especially at the grass-roots, rural-community level. Our preliminary estimates indicate that the biomass pellet sector currently generates over 5 million person-months in the construction of BPBP plants and over 200,000 full-time employments in the operation of biomass power plants and in the production of biomass pellets. Agricultural and forestry residues have very important long-term potential in India. Biomass pellets are important for socio-economic development. Hence, central and provincial governments and institutions should start working on specific strategies and policies to support the exploitation of agricultural and forestry residues for energy purposes.

Ultimately, transportation distance is a decisive factor in the economics of BPBP plants. States should assess their respective potential for the production of agricultural and forestry residues, determine their targets and ambitions for biomass-based electricity generation, and then devise strategies for the use of pelletized versus non-pelletized biomass in electricity generation plants.

## Electronic supplementary material

ESM 1 (DOCX 274 kb)

## Footnotes

1 Indian coals have the high ash content (ranging from 40 to 50%), high moisture content (4–20%), low sulfur content (0.2–0.7%), and low calorific values (between 10.5 and 20.9 MJ/kg) (IEA 2002; Chikkatur et al. 2009).

2 Out of 1400 MW, installed capacity of grid-connected biomass power projects were 998 MW, installed capacity of off-grid biomass cogeneration (non-bagasse) projects were 274 MW, and installed capacity of biomass gasification projects were 128 MW in 2010–11.

3 After taking into account the fraction of agricultural residues used for fodder, fuel, and other competitive applications.

4 Here, surplus implies residue available after allocating for livestock, soil, and other competing applications.

5 As per the recent Agreement at the 21st Conference of the Parties (COP-21) in Paris, world leaders have agreed to attempt limiting the temperature increase to 1.5 °C.
