# Supplementary material for: Biomass pellets for power generation in India: a techno-economic evaluation
Source: Environ Sci Pollut Res Int. 2018 Aug 24;25(29):29614–32. doi: 10.1007/s11356-018-2960-8 (PMC6153682; doi:10.1007/s11356-018-2960-8)
Supplement: Supplementary file 1 — (DOCX 274 kb) [file 11356_2018_2960_MOESM1_ESM.docx]

**BIOMASS PELLETS FOR POWER GENERATION IN INDIA:**

**A TECHNO-ECONOMIC EVALUATION**

Pallav Purohit^*^^[[1]](#footnote-1)^, Vaibhav Chaturvedi^**^

^*^International Institute for Applied Systems Analysis (IIASA), Laxenburg, Austria

^**^Council on Energy, Environment and Water (CEEW), New Delhi, India

## S.1 Agricultural residue availability in India

Agriculture plays a vital role in India’s economy. According to the new series of national income released by the Central statistical Organization (CSO), at 2011-12 prices the share of agriculture in total GDP was 18% in 2013-14 (GoI, 2015). Over 58% of the rural households depend on agriculture as their principal means of livelihood (IBEF, 2015). Of India’s total geographic area of 328 million hectares (Mha), the net cropped area accounts for approximately 43%, and it appears that the net cropped area has stabilised at approximately 140 Mha since 1970 (Ravindranath et al., 2005). However, the gross cropped area, which measures multiple crops grown per year, increased from 132 Mha in 1950–51 to approximately 195 Mha in 2008–09. There are two main growing seasons in India, namely *Kharif* (monsoon season in the southwest) and *Rabi* (monsoon season in the north-east). The gross cropped area includes land that produces multiple crops in the same year (usually two crops), mainly on irrigated land. The net irrigated area has increased substantially in the last few decades, from 21 Mha in 1950–51 to 63 Mha in 2008–09. Rice and wheat are the dominant crops, together accounting for 41% of the cropped area, while pulses, oil seeds, and other commercial crops account for 13.8%, 15.9% and 10.2%, respectively. Table 1 presents the area and production of different crops in India for the base year 2010 taken from (MoA, 2012). We use a simple linear regression model to estimate the area and production of major crops in the near future. Figure S.1 presents the time variation of area and production for rice. It can be noted that rice production increased from 20.6 million tonne (Mt) in 1950-51 to 105.3 Mt in 2011-12. During the same period, the rice yield increased by a factor of 3.6, whereas the area under rice cultivation increased from 31 million hectare (Mha) to 44 Mha.


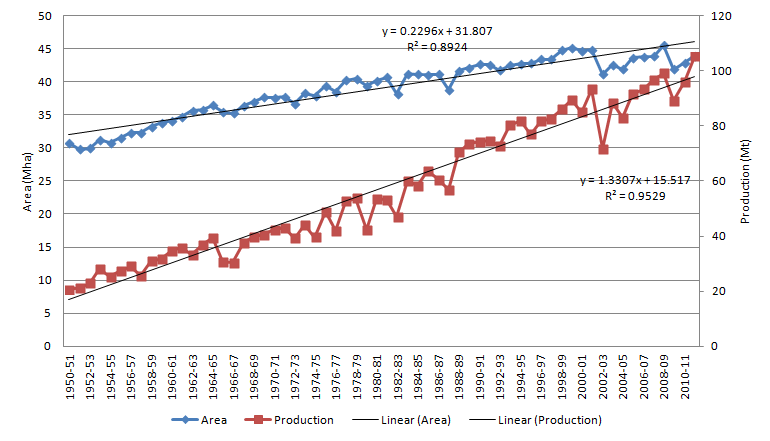


**Figure S.1: Time variation of area and production of rice in India**

Figure S.2 presents the time variation of the area and production for wheat. Wheat production increased from 6.5 Mt in 1950-51 to 95 Mt in 2011-12. During the same period, the yield increased by a factor of 4.8, whereas the area under wheat cultivation increased from 9.8 Mha to 29.9 Mha.


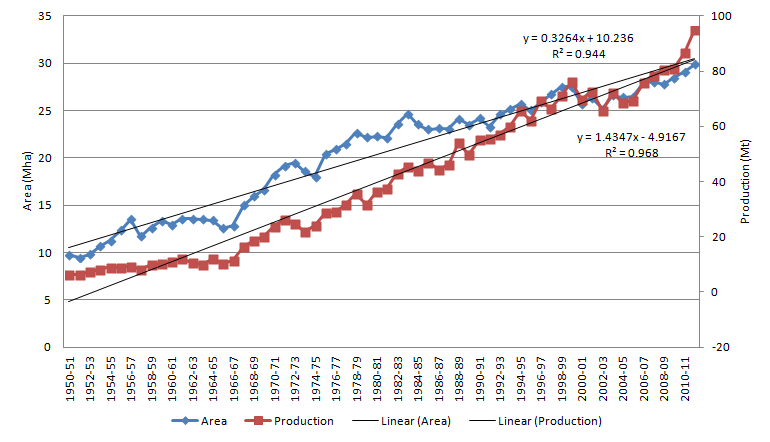


**Figure S.2: Time variation of area and production of wheat in India**

Figure S.3 presents the time variation of area and production for maize. Maize production increased from 1.73 Mt in 1950-51 to 21.76 Mt in 2011-12. During the same period, the maize yield increased by a factor of 4.53, whereas the area under maize cultivation increased from 3.16 Mha to 8.78 Mha.


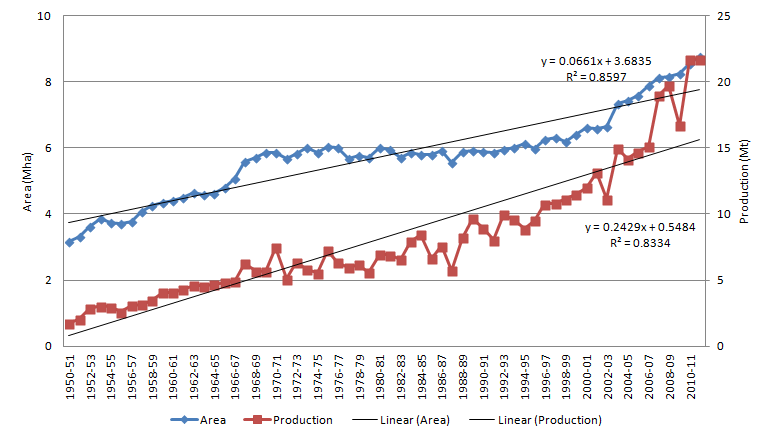


**Figure S.3: Time variation of area and production of maize in India**

Figure S.4 presents the time variation of area and production for lentil in India. Lentil production increased from 0.37 Mt in 1970-71 to 0.95 Mt in 2010-11. During the same period, the lentil yield increased by a factor of 1.2, whereas the area under maize cultivation increased from 0.75 to 1.6 Mha.


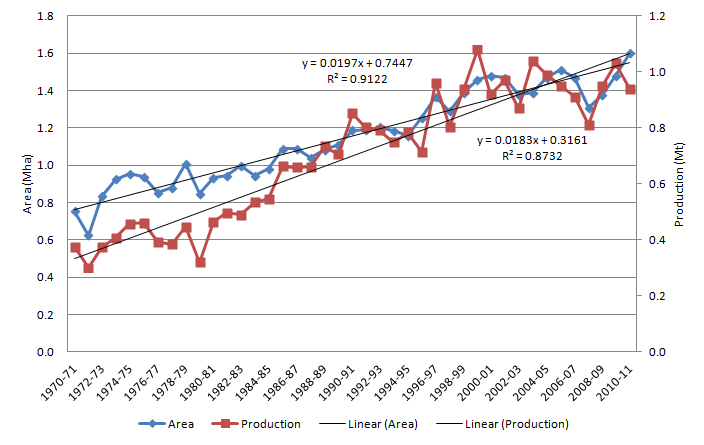


**Figure S.4: Time variation of area and production of lentil in India**

Figure S.5 presents the time variation of area and production for sugarcane in India. Sugarcane production increased from 57.1 Mt in 1950-51 to 342.4 Mt in 2010-11. During the same period, the sugarcane yield increased by a factor of 2.1, whereas the area under sugarcane cultivation increased from 1.71 Mha to 4.88 Mha.


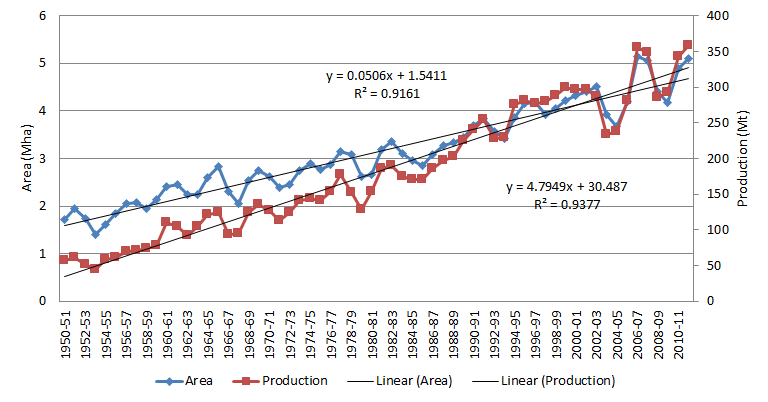


**Figure S.5: Time variation of area and production of sugarcane in India**

Figure S.6 presents the time variation of area and production for coconut in India. Coconut production increased from 35.8 million nuts in 1950-51 to 157.5 million nuts in 2010-11. During the same period, the coconut yield increased by a factor of 1.4, whereas the area under coconut cultivation increased from 0.62 Mha to 1.9 Mha. Similarly, the area and production of other crops is projected based on the data from 1950-51 to 2011-12 (MoA, 2012).


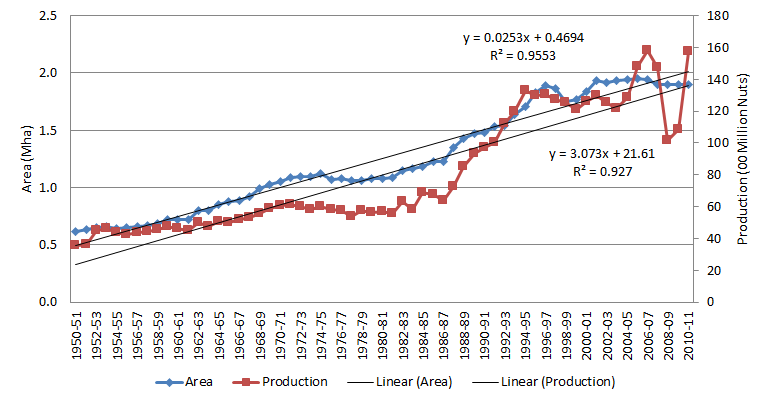


**Figure S.6: Time variation of area and production of coconut in India**

## References

GoI. 2015. Economic Survey, 2014-15. Office of the Registrar General and Census Commissioner, Government of India (GoI), New Delhi.

IBEF. 2015. Indian Agriculture Industry: an overview. India Brand Equity Foundation (IBEF), New Delhi (See: <http://www.ibef.org/industry/agriculture-india.aspx> Accessed on 28th December 2015).

Ravindranath, N.H., Somashekar, H.I., Nagaraja, M.S., Sudha, P., Sangeetha, G., Bhattacharya, S.C., Salam, P.A. 2005. Assessment of Sustainable Non-Plantation Biomass Resources Potential for Energy in India. Biomass and Bioenergy, 29 (3): 178-190.

1. Corresponding author: Tel: +43-2236807-336; Fax: +43-2236807-533; E-mail: [purohit@iiasa.ac.at](mailto:purohit@iiasa.ac.at) [↑](#footnote-ref-1)
